# African Ancestry Is a Predictor Factor to Secondary Progression in Clinical Course of Multiple Sclerosis

**DOI:** 10.5402/2012/410629

**Published:** 2012-11-25

**Authors:** Claudia Cristina Ferreira Vasconcelos, Gutemberg Augusto Cruz dos Santos, Luiz Claudio Thuler, Solange Maria Camargo, Regina Maria Papais Alvarenga

**Affiliations:** Departamento de Neurologia, Hospital Universitário Gaffrée e Guinle, Rua Mariz e Barros 775, 2° andar, Maracanã-Tijuca, 20270-004 Rio de Janeiro, RJ, Brazil

## Abstract

*Background*. Studies on the clinical course of multiple sclerosis have indicated that certain initial clinical factors are predictive of disease progression. Regions with a low prevalence for disease, which have environmental and genetic factors that differ from areas of high prevalence, lack studies on the progressive course and disabling characteristics of the disease. *Objective*. To analyse the long-term evolution to the progressive phase of the relapsing-remitting multiple sclerosis and its prognosis factors in mixed population. *Methods*. We performed a survival study and logistic regression to examine the influence of demographic and initial clinical factors on disease progression. Among 553 relapsing-remitting patients assisted at a Brazilian reference centre for multiple sclerosis, we reviewed the medical records of 150 patients who had a disease for ten or more years. *Results*. African ancestry was a factor that conferred more risk for secondary progression followed by age at the onset of the disease and the number of relapses in the year after diagnosis. A greater understanding of the influence of ancestry on prognosis serves to stimulate genetics and pharmacogenomics research and may clarify the poorly understood neurodegenerative progression of MS.

## 1. Introduction

The current understanding of multiple sclerosis (MS) as a progressive and long-term disabling disease has been based on studies of clinical course of the disease in areas of high disease prevalence [1, 2].

Although Weinshenker et al. (1989) have reported that approximately 50% of relapsing-remitting cases required an unilateral support to walk within an average of 15 years [1], recent data has suggested a better long-term prognosis [3, 4]. The age of onset, the number of relapses early, and the interval between initial relapses have been identified as predictive factors for the development of long-term disability outcomes [2, 5, 6]. 

An increasing number of studies have identified the population ancestry as a risk factor, not only for susceptibility to MS but also for clinical progression. Disease outcomes have been shown to be poorer in patients with African ancestry [7–11]. In Latin America, which has environmental and genetic factors that differ from those high disease prevalence areas, a worse prognosis for African descendants with the primary progressive form of MS (Primary Progressive Multiple Sclerosis (PPMS)) was already identified [11], but there is a lack of studies on the clinical progression and long-term disability outcomes in the relapsing-remitting form of multiple sclerosis (RRMS) [12].

The aim of this study was to describe and analyse the secondary progression of RRMS in a tropical region with higher proportions of African descents, taking into consideration the influence of clinical characteristics and demographic as ancestry.

## 2. Methods

### 2.1. Population and Data Collection

We conducted a study of survival on a subset of patients with RRMS [12] long term followed at the Hospital da Lagoa in Rio de Janeiro, Brazil. Care for patients with MS was established at this hospital in 1990, which is currently one of the referral centres for demyelinating diseases in the state of Rio de Janeiro. Care for patients suspected of having MS occurs through spontaneous demand or through a referral from health services in the southeast region of the country. By December 2010, there were 623 registered patients with MS, and demographic information and data from clinical visits from RRMS patients with the duration of the disease of ten or more years were extracted from the Brazilian Software Database for Multiple Sclerosis Research in Tropical Countries (SIAPEM). PPMS patients were not included due to the presence of progression since the onset of the disease which could generate bias in the measurement of time to the beginning of disability progression, the primary endpoint of this study. Additionally parameters like number of relapses and the time interval between them would not be possible to assess in primary progressive MS group.

### 2.2. Inclusion Criteria

From the registry of all patients with RRMS according to McDonald et al. Criteria [13], 150 cases with the duration of the disease of ten or more years and regular monitoring data were included.

### 2.3. Evaluation Measures

Clinical onset was defined as the year of the first relapse. The observation period concluded with the last recorded evaluation in or before December 31, 2010.

Information on relapses, recovery, disability, and progression scores was obtained from the records of follow-up visits.

Cases with an initial optic spinal manifestation were reviewed and specific criteria [14] for NMO were applied to exclude the possibility of neuromyelitis optica (NMO).

Relapse was defined as the presence of acute neurological symptoms or worsening of preexisting symptoms for longer than 24 hours and followed by partial or complete remission [13, 15].

Initial clinical manifestations were categorised by functional system (FS): pyramidal, cerebellar, sensory, visual, brainstem, sphincter, and psychiatric. Disability scores were measured with the Kurtzke Expanded Disability Status Scale (EDSS) [16].

The clinical course was classified as benign if the EDSS score was 3 or less after ten years of disease [17] and classified as malignant if the EDSS score was 6 or more after five years of disease [18].

The secondary progression (progressive phase onset of the disease or secondary progressive multiple sclerosis (SPMS)) was defined as a maintained increase in EDSS score not attributed to a relapse that continued for six months or longer with no improvement or progressive worsening with each assessment.

The time to secondary progression was investigated in relation to the following factors: gender (male versus female); ancestry: (African descent—if there were blacks in the family as far back as the third generation versus white); age at onset of disease (less than 30 years versus 30 years or more); type (pyramidal, cerebelar, sensory, brain stem, visual, sphincter, and mental); number of initial neurological manifestations (one versus two or greater than two); number of relapses in the first year of disease (until two versus more than two); first interattack interval (until two years versus more than two years); recovery from the first relapse: residual deficits absent versus present; immunosuppressive or immunomodulatory therapy: used versus not used.

### 2.4. Statistical Methods

The *Statistical Package for the Social Sciences* (SPSS) 13.0 for Windows was used to perform the analyses. Quantitative variables were represented by frequency (%), and tests of skewness and kurtosis were applied to continuous variables to confirm normal distribution and enable the use of the means ± standard deviation. The Mann-Whitney test was applied for the comparison of continuous variables and Pearson Chi-squared or Fisher's exact tests were used for categorical variables. Odds ratio was also calculated to identify factors associated with the long-term disability outcomes and faster onset of progression. Kaplan-Meier curves with log-Rank tests were used to assess the cumulative risk to the progressive phase according to demographic and clinical data. In the survival analysis, it was calculated the median time in years to reach the secondary progression. Cox multivariate analysis was performed to determine the variables independently associated with a faster onset of progression. Only variables with *P* values of less than 0.05 in the bivariate analysis were included in the multivariate model. The *Forward Logistic Regression *(Forward LR) was applied in the Cox regression analysis. Only Kaplan-Meier curves of statistically significant factors in Cox regression were represented graphically.

Statistical significance was set at *P* < 0.05.

## 3. Results

Of 623 patients with MS in the registry, 553 (88%) were diagnosed with RRMS [12, 13]. Of these cases, 150 (27%) had the disease for ten or more years and were included in the study. The average duration of disease for this group was 19.7 ± 9.1 years (range: 10–53 years).

### 3.1. General Characteristics of the Studied Population

Demographic and clinical characteristics for the cases are represented in Table 1. The data are summarised for the entire group of patients and are broken down by gender and ancestry.

#### 3.1.1. Demographics

The majority of patients were female (79%) and Caucasian (78%). In most patients, the age of disease onset was between the third and fourth decades of life.

#### 3.1.2. Initial Clinical Conditions

Most patients (82%) had one relapse in the first year of disease, and of those most experienced a full recovery (83%).

The initial clinical condition was characterised by the involvement of one functional system in two-thirds of the patients, and sensory manifestations were the most common. Stratification by gender and ethnicity showed a trend towards predominance of motor symptoms in male patients (*P* = 0.05).

The time interval between the first and second relapses was no more than two years in more than half of the patients.

No significant differences in initial clinical characteristics were noted when controlling for gender and ethnicity.

### 3.2. Evolution: Benign Course; Severe or “Malignant” Course; Progression

Approximately 40% of patients, with a mean age of 44 years and an average duration of disease of 13 years, advanced to the progressive form of MS. A higher frequency of men (45.2%) and of African descent patients (51.5%) progressed to the secondary progressive form, without statistical significance. No significant difference was observed in the mean age at the onset of progression with respect to gender or ethnicity.

Approximately 80% of patients at ten years of the disease had benign MS. A larger number of Caucasians were significantly more often (85%, IC95%: 78.2%–92.0%; *P* = 0.002) to have benign MS than African descent patients (60,6%; IC95%: 43.4%–76%), while the latter more often to reach EDSS score of 6 (12.1%; IC95%: 3,9%–26.7%; *P* = 0.007) within a time interval of five years or less (“malignant” evolution) than Caucasians (1.7%; IC95%: 0.3%–5.5%).

Disability was significantly different between groups of ancestry: EDSS median scores were higher in African descent patients than in non-African descent at five years of the disease (1.9 versus 0, *P* = 0.002), at ten years of the disease (2.0 versus 1.0, *P* = 0.01) and at the last follow-up visit (5.5 versus 3.0, *P* = 0.016).

### 3.3. Diagnosis and Treatment

On average, patients waited five to six years before receiving a diagnosis. The shortest period between disease onset and diagnosis was one month and the longest was 29 years.

Over 70% of patients (111/150) were treated with disease-modifying drugs (DMDs) for at least 3 months, including immunomodulators (102/111) and immunosuppressants (9/111). The average time before initiating therapy was greater than ten years in 54% of the patients and ranged from one to 38 years, with no significant differences with regard to gender. African descent and non-African descent patients were diagnosed and treated after medians of time very close (Table 2); no significant differences were noted between the groups.

### 3.4. Influence of Demographic and Clinical Factors on Time to Progression

#### 3.4.1. Time to Reach the Progressive Phase of Disease, according to Demographics and Clinical Factors

There was no statistically significant difference between the genders with respect to the amount of time before the progression of disease (*P* = 0.31).

African descent patients reached the progressive phase more quickly than non-African descent (11.0 years versus 15.0 years, *P* = 0.006).

In patients who developed MS when they were 30 years and older, the time taken to reach the secondary progression was more rapid than in patients who developed MS when they were younger (14.0 years versus 17.0 years, *P* = 0.004).

Similarly, patients with first interattack interval less than two years reached the secondary progression of the disease more rapidly (13.0 versus 17.5 years, *P* < 0.0001).

Patients who experienced first relapse that did not fully remit presented more short time to progression (15.0 years versus 11.0 years, *P* = 0.04).

The time to reach disease progression was significantly less in patients who had more than one relapse in the first year of disease (11.7 versus 17.6 years *P* < 0.001).

#### 3.4.2. Risk for Progression

**Figure 1:**
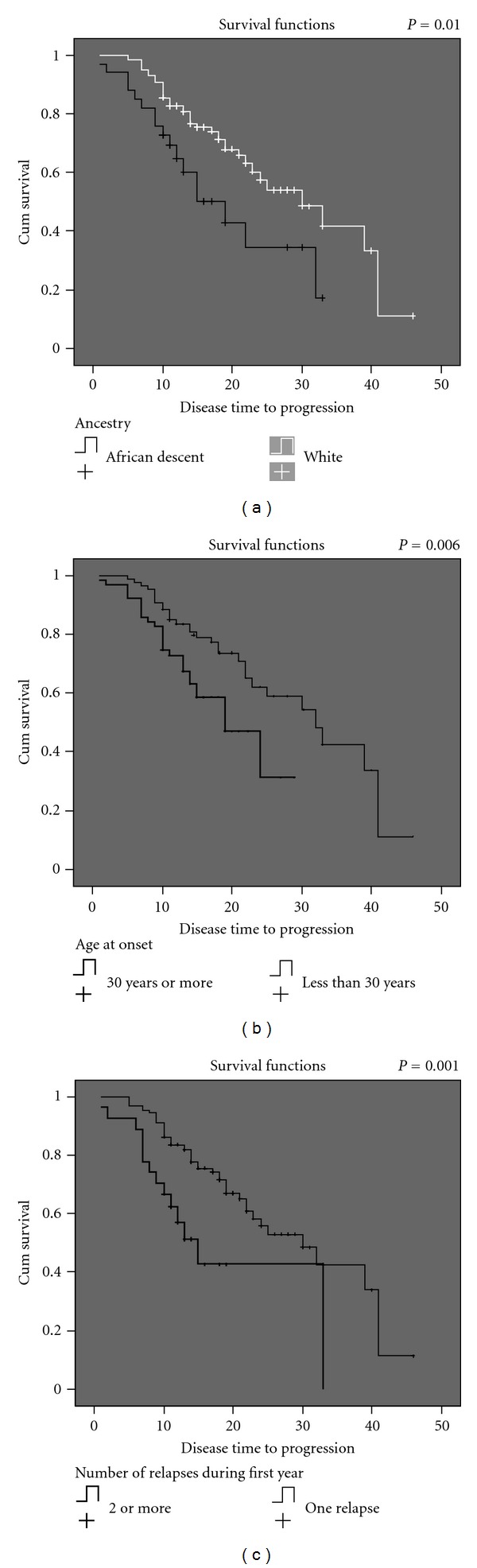
Survival curves (a), (b), and (c) represent the median time to reach progression phase, respectively, according to the ancestry, age at onset of the disease, and the number of relapses in the first year of the disease.

Cox logistic regression was performed to assess the effect of ancestry and other initial clinical factors on time to the progressive phase onset of the disease (Table 3). Patients of African descent, patients older than 30 years of age, and patients who experienced more than one relapse in the first year of disease had a higher risk for progression. Figure 1 shows the time-to-onset curves of the progressive phase of MS according to ancestry, age at onset, and number of relapses during the first year of the disease.

### 3.5. Influence of Treatment on the Evolution of Disability and Progression

For the patients who reached the secondary progressive phase of MS, the use of DMD had no significant effects on median times in the Kaplan-Meier curves or on the risk for progression (25.5 years versus 29 years, *P* = 0.08).

## 4. Discussion

Considering the scarcity of longitudinal studies and prognosis in Latin America, the current study is the first to analyze the influence of demographic and clinical factors on the survival of RRMS patients of mixed ethnicity. The time to conversion in secondary progressive MS was analysed in 150 patients with more than 10 years of illness which remained in a followup on a reference centre for the treatment of MS. Kremenchutzky et al. (2006) have suggested that the probability of and time to progression have been poorly explored despite having profound relevance [19]. Additionally it is important to note that various therapeutic options currently available have not been able to prevent the progressive phase of the disease or disability yet [20–22]. 

The selection of cases with more than ten years of evolution was provided for sufficient time to observe both benign and malignant trajectories of the disease course. Moreover, this time frame would theoretically minimise the possible influence of DMD because the distribution of these drugs in our country began in the last decade. This is supported by the average time of 11 years before beginning DMD treatment. Another observation is that the average time to diagnosis of MS and to the initiation of treatment did not differ between African descendants and Caucasians, which suggests that the potential influences of socioeconomic factors related to race did not affect our results. The use of DMD did not significantly affect the time to disease progression. The results obtained from clinical trials with immunomodulatory [20–22] and immunosuppressive drugs (cladribine) [23] and monoclonal antibodies (alemtuzumab) [24] have not clearly shown that a reduction in relapses slows disease progression. Immune-mediated inflammation is the predominant pathogenic mechanism in the initial phases of MS [25]. The optimal therapeutic window targets this period of inflammation.

Anatomopathological studies indicate that the biological mechanisms responsible for neurodegeneration and disability are distinct from those that cause inflammation and relapses [25, 26]. The occurrence of relapses was initially implicated as one of the main determinants of degeneration and progression [1, 2, 6, 27, 28]. According to Compston the progression of MS is due to axonal loss initiated and maintained by the inflammatory response in individuals susceptible to neurodegeneration [29]. Other authors consider the progressive phase of the disease to be an age-dependent degenerative process [19]. The time to progression and time to disability would be predetermined, with no influence of relapses or the initial course of the disease [30]. It remains unclear whether the accumulation of relapses and long-term disability are causally related [31]. The predictive value of the rate of early relapses on disease progression seems to be weaker once the progressive course of the disease is initiated [5, 6]. The results obtained in our study confirm previous observations that a higher rate of relapses in the initial phase of MS, especially in the first year, could increase the risk for progression [31, 32]. However, once the progressive phase is reached, the influence of relapses disappears [32].

A difference in the speed to long-term disability outcomes between men and women has been identified, with a worse prognosis for male patients [1, 2, 6]. However, in our results the time interval for the onset of progression did not differ significantly between genders. This finding strengthens the observation made by Leray et al. that the strength of the identified risk factors could be limited to early stages of the disease [32].

Later age at disease onset is another factor that has been described as a risk factor for progression [4, 30, 32–35]. According to our results, patients who were 30 years or older when they developed the disease had an increased risk for moderate disability and progressed after a shorter time interval, supporting the possibility of a degenerative age-dependent component [19].

The presence of residual deficits after the first neurological manifestation [32] and a shorter time interval between the first and second relapses were identified as predictive of a worse course [1, 3, 6–8, 32]. Our results confirm these findings. In the multivariate analysis, these factors lost strength when they were analysed together with the number of relapses in the first year, older age, and African ancestry. African ancestry has been associated with a worse prognosis for various diseases [36–39]. Genetic factors linked to ethnicity may be related to the initiation and evolution of MS. Studies of African Americans analysed relapsing-remitting and initially progressive forms of disease in conjunction [7–10]. The Brazilian study, which assessed the evolution of PPMS in a multiracial population, proved that African ancestry increased disease severity even in the form of the disease with the worst prognosis [11]. In turn, the results of our study, which was conducted in the same population, but with RRMS, also indicate that African ancestry is a strong predictive factor for worse outcomes. In addition significantly higher median EDSS scores at five and ten years and at the last followup were observed in the African descent group which were more likely to have malignant forms of the disease than Caucasians.

The poorer outcomes in individuals of African ancestry could suggest the participation of non-HLA genes; as an example is the polymorphisms in *TNFRSF1A,* that could also contribute to a more degenerative course of MS [40].

## 5. Conclusion

The intervals of time from the onset of the disease until the onset of the progressive phase were influenced by initial clinical characteristics and mainly by ancestry, the latter being a biological variable intrinsic to the patient, not the disease. Both conditions, demographic and clinical, are present at the onset of the disease and should be considered together with the therapeutic window in intervention strategies. The recognition of the role of ancestry on prognosis also serves to stimulate genetics and pharmacogenomics research that may clarify the poorly understood neurodegenerative component of MS.

## Figures and Tables

**Table 1:** Initial clinical factors and progression characteristics according to gender and ethnicity.

Characteristics	General	Female	Male	African descent	White
*N* (%)	150	119 (79.3)	31 (20.7)	33 (22.0)	117 (78.0)
Age at disease onset					
Mean ± SD	29.3 ± 9.3	28.8 ± 9.5	30.3 ± 8.8	31.1 ± 9.4	28.2 ± 9.3
Involved functional systems (FS) at onset					
1 FS (%)	104 (69.3)	86 (72.3)	18 (58.1)	23 (69.7)	81 (69.2)
≥2 FS (%)	46 (30.7)	33 (27.7)	13 (41.9)	10 (30.3)	36 (30.8)
Initial symptoms of MS (%)					
Pyramidal	55 (36.3)	39 (32.8)	16 (51.6)	14 (42.4)	41 (35.0)
Cerebellar	22 (14.7)	15 (12.6)	7 (22.6)	3 (9.1)	19 (16.2)
Visual	23 (15.3)	19 (16.0)	4 (12.9)	4 (12.1)	19 (16.2)
Sensory	67 (44.7)	52 (43.7)	15 (48.4)	17 (51.5)	50 (42.7)
Brainstem	33 (22.0)	28 (23.5)	5 (16.1)	6 (18.2)	27 (23.1)
Sphincter	6 (4.0)	4 (3.4)	2 (6.5)	1 (3.0)	5 (4.3)
Psychiatric	2 (1.3)	2 (1,7)	0	1 (3.0)	1 (0.9)
First interval between exacerbations, years					
Short (≤2 years)	85 (56.7)	64 (53.8)	21 (67.7)	19 (57.6)	66 (56.4)
Long (>2 years)	65 (43.3)	55 (46.2)	10 (32.3)	14 (42.4)	51 (43.0)
Recovery from 1st exacerbation					
Complete recovery	124 (82.7)	100 (84.0)	24 (77.4)	24 (72.7)	100 (85.5)
Incomplete recovery	26 (17.3)	19 (15.9)	7 (22.6)	9 (27.2)	17 (14.5)
Relapses in the first year of disease (%)					
1 exacerbation	123 (82.0)	98 (82.4)	25 (80.6)	24 (72.7)	99 (84.6)
≥2 exacerbations	27 (18)	21 (17.6)	6 (19.4)	9 (27.3)	18 (15.4)
Benign disease (%)					
Yes	120 (80.0)	97 (81.5)	23 (74.2)	20 (60.6)	100 (85.5)*
No	80 (20)	22 (18.5)	8 (25.8)	13 (39.4)	17 (14.5)
Malignant disease (%)					
Yes	6 (4.0)	4 (3.4)	2 (6.5)	4 (12.1)*	2 (1.7)
No	144 (96)	115 (96.6)	29 (93.5)	29 (87.9)	115 (98.3)
Secondary progression (%)					
Yes	59 (39.3)	45 (37.8)	14 (45.2)	17 (51.5)	42 (35.9)
No	91 (60.7)	74 (62.2)	17 (54.8)	16 (48.5)	75 (64.1)

FS: functional system of Kurtzke's EDSS scale; benign disease: at 10 years of disease EDSS 3 or less; malign disease: at 5 years of disease EDSS 6 or more; *P < 0.05.

**Table 2:** Times to diagnosis and treatment according to ancestry.

Ancestry	Time of disease to diagnosis	Time of disease to start treatment
White		
Median	4.0	10.0
Minimum	0.10	0.60
Maximum	29.0	38.0
Afro		
Median	3.0	8.0
Minimum	0,50	1,00
Maximum	28.0	41.0
	*P* = 0.73	*P* = 0.66
Total		
Median	3.5	10.0
Minimum	0,10	0.60
Maximum	29.0	41.0

**Table 3:** Multiple Cox regression survival analysis: risk of reaching progression according to ethnicity and clinical features at the onset of disease.

Outcome/variable	Risk	95% CI	*P* value
Progression			
African descent	2.0	1.1–3.4	0.02
30 years of age or older at onset	2.0	1.1–3.3	0.02
2 or more exacerbations during the first year	2.3	1.2–4.3	0.009

## References

[1] Weinshenker BG, Bass B, Rice GPA (1989). The natural history of multiple sclerosis: a geographically based study. I. Clinical course and disability. *Brain*.

[2] Weinshenker BG, Bass B, Rice GPA (1989). The natural history of multiple sclerosis: a geographically based study. 2. Predictive value of the early clinical course. *Brain*.

[3] Pittock SJ, Mayr WT, McClelland RL (2004). Disability profile of MS did not change over 10 years in a population-based prevalence cohort. *Neurology*.

[4] Tremlett H, Paty D, Devonshire V (2006). Disability progression in multiple sclerosis is slower than previously reported. *Neurology*.

[5] Confavreux C, Vukusic S, Moreau T, Adeleine P (2000). Relapses and progression of disability in multiple sclerosis. *The New England Journal of Medicine*.

[6] Confavreux C, Vukusic S, Adeleine P (2003). Early clinical predictors and progression of irreversible disability in multiple sclerosis: an amnesic process. *Brain*.

[7] Weinstock-Guttman B, Jacobs LD, Brownscheidle CM (2003). Multiple sclerosis characteristics in African American patients in the New York State Multiple Sclerosis Consortium. *Multiple Sclerosis*.

[8] Cree BAC, Khan O, Bourdette D (2004). Clinical characteristics of African Americans vs Caucasian Americans with multiple sclerosis. *Neurology*.

[9] Naismith RT, Trinkaus K, Cross AH (2006). Phenotype and prognosis in African-Americans with multiple sclerosis: a retrospective chart review. *Multiple Sclerosis*.

[10] Jeannin S, Bourg V, Berthier F, Lebrun C (2007). Phenotypical aspects and clinical course of multiple sclerosis in 76 patients with a North African ethnic background followed at the Nice University Hospital. *Revue Neurologique*.

[11] Vasconcelos CCF, Santos Thuler LC, Dos Santos GAC (2010). Differences in the progression of primary progressive multiple sclerosis in Brazilians of African descent versus white Brazilian patients. *Multiple Sclerosis*.

[12] Lublin FD, Reingold SC (1996). Defining the clinical course of multiple sclerosis: results of an international survey. *Neurology*.

[13] McDonald WI, Compston A, Edan G (2001). Recommended diagnostic criteria for multiple sclerosis: guidelines from the International Panel on the Diagnosis of Multiple Sclerosis. *Annals of Neurology*.

[14] Wingerchuk DM, Lennon VA, Pittock SJ, Lucchinetti CF, Weinshenker BG (2006). Revised diagnostic criteria for neuromyelitis optica. *Neurology*.

[15] Poser CM, Paty DW, Scheinberg L (1983). New diagnostic criteria for multiple sclerosis: guidelines for research protocols. *Annals of Neurology*.

[16] Kurtzke JF (1983). Rating neurologic impairment in multiple sclerosis: an expanded disability status scale (EDSS). *Neurology*.

[17] Kantarci OH, Weinshenker BG (2005). Natural history of multiple sclerosis. *Neurologic Clinics*.

[18] Perini P, Tagliaferri C, Belloni M, Biasi G, Gallo P (2001). The HLA-DR13 haplotype is associated with "benign" multiple sclerosis in northeast Italy. *Neurology*.

[19] Kremenchutzky M, Rice GPA, Baskerville J, Wingerchuk DM, Ebers GC (2006). The natural history of multiple sclerosis: a geographically based study 9: observations on the progressive phase of the disease. *Brain*.

[20] Paty DW, Li DKB (1993). Interferon beta-1b is effective in relapsing-remitting multiple sclerosis. II. MRI analysis results of a multicenter, randomized, double-blind, placebo- controlled trial. *Neurology*.

[21] Johnson KP, Brooks BR, Cohen JA (1995). Copolymer 1 reduces relapse rate and improves disability in relapsing-remitting multiple sclerosis: results of a phase III multicenter, double- blind, placebo-controlled trial. *Neurology*.

[22] European Study Group on interferon beta-1b in secondary progressive MS (1998). Placebo-controlled multicentre randomised trial of interferon *β*-1b in treatment of secondary progressive multiple sclerosis. *The Lancet*.

[23] Rice GPA, Filippi M, Comi G (2000). Cladribine and progressive MS: clinical and MRI outcomes of a multicenter controlled trial. Cladribine MRI Study Group. *Neurology*.

[24] Coles AJ, Wing MG, Molyneux P (1999). Monoclonal antibody treatment exposes three mechanisms underlying the clinical course of multiple sclerosis. *Annals of Neurology*.

[25] Trapp BD, Nave KA (2008). Multiple sclerosis: an immune or neurodegenerative disorder?. *Annual Review of Neuroscience*.

[26] DeLuca GC, Williams K, Evangelou N, Ebers GC, Esiri MM (2006). The contribution of demyelination to axonal loss in multiple sclerosis. *Brain*.

[27] Lublin FD, Baier M, Cutter G (2003). Effect of relapses on development of residual deficit in multiple sclerosis. *Neurology*.

[28] Tremlett H, Yousefi M, Devonshire V, Rieckmann P, Zhao Y (2009). Impact of multiple sclerosis relapses on progression diminishes with time. *Neurology*.

[29] Compston A (2006). Making progress on the natural history of multiple sclerosis. *Brain*.

[30] Confavreux C, Vukusic S (2006). Age at disability milestones in multiple sclerosis. *Brain*.

[31] Scalfari A, Neuhaus A, Degenhardt A (2010). The natural history of multiple sclerosis, a geographically based study 10: relapses and long-term disability. *Brain*.

[32] Leray E, Yaouanq J, Le Page E (2010). Evidence for a two-stage disability progression in multiple sclerosis. *Brain*.

[33] Phadke JG (1990). Clinical aspects of multiple sclerosis in north-east Scotland with particular reference to its course and prognosis. *Brain*.

[34] Koch M, Kingwell E, Rieckmann P (2010). The natural history of secondary progressive multiple sclerosis. *Journal of Neurology, Neurosurgery and Psychiatry*.

[35] Stankoff B, Mrejen S, Tourbah A (2007). Age at onset determines the occurrence of the progressive phase of multiple sclerosis. *Neurology*.

[36] Howard G, Howard VJ (2000). Ethnic disparities in stroke: The scope of the problem. *Ethnicity and Disease*.

[37] Arbuckle MR, James JA, Dennis GJ (2003). Rapid clinical progression to diagnosis among African-American men with systemic lupus erythematosus. *Lupus*.

[38] De Jesus MA, Fujita M, Kim KS (2003). Retrospective analysis of breast cancer among young African American females. *Breast Cancer Research and Treatment*.

[39] Govindarajan R, Shah RV, Erkman LG, Hutchins LF (2003). Racial differences in the outcome of patients with colorectal carcinoma. *Cancer*.

[40] Johnson BA, Wang J, Taylor EM (2010). Multiple sclerosis susceptibility alleles in African Americans. *Genes and Immunity*.

